# Supplementary material for: A Portable, Negative-Pressure Actuated, Dynamically Tunable Microfluidic Droplet Generator
Source: Micromachines (Basel). 2022 Oct 25;13(11):1823. doi: 10.3390/mi13111823 (PMC9697964; doi:10.3390/mi13111823)
Supplement: Supplementary file 1 [file micromachines-13-01823-s001.zip › micromachines-1960590-supplementary.pdf]

Supplementary

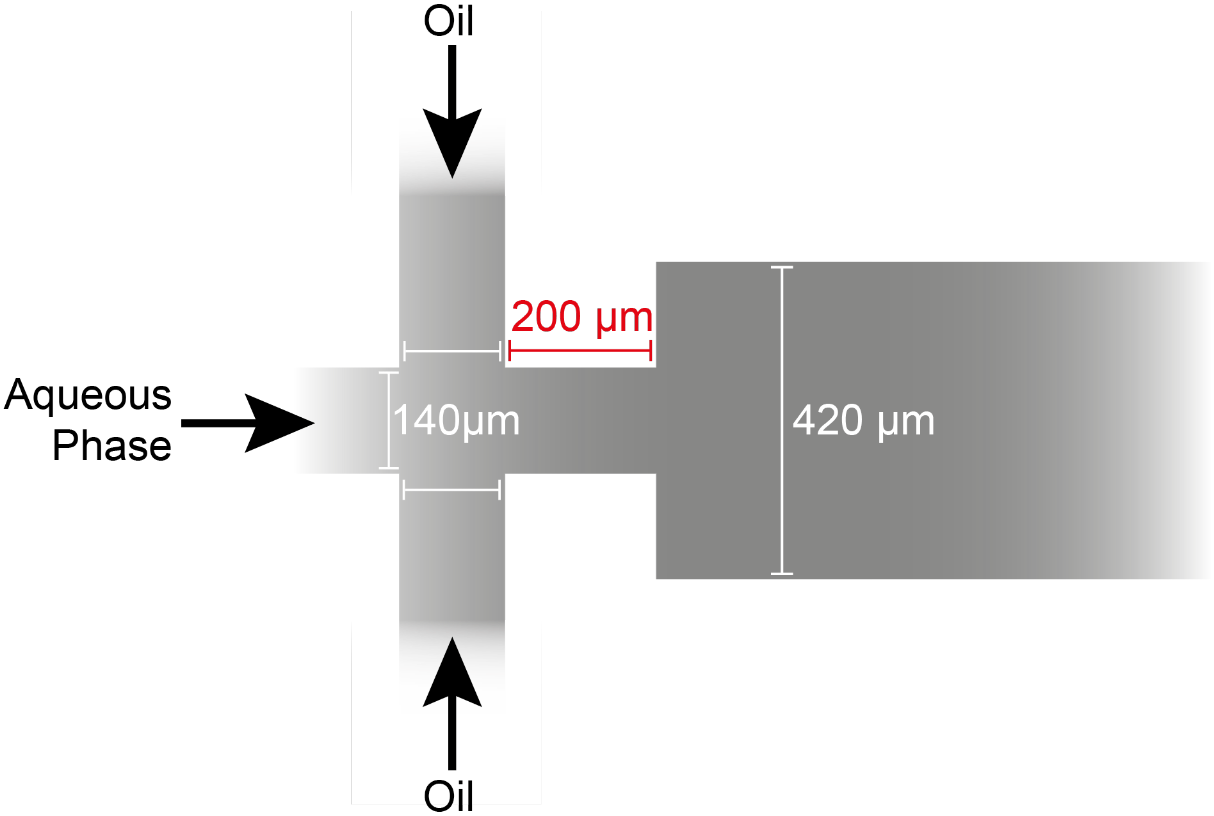

Supplemental Figure S1. Schematic two-dimensional depiction of the nozzle region with the relevant dimensions. The channel depth is 175 µm.

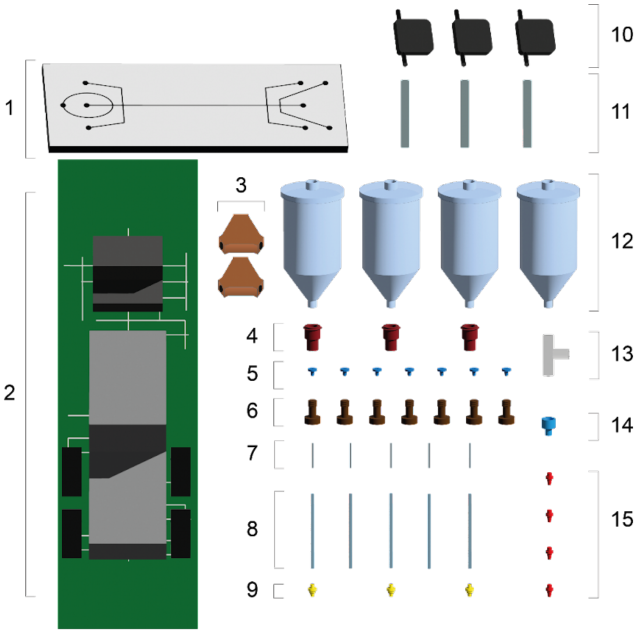

	Part	Manufacturer
1	Microfluidic Chip	microfluidic ChipShop
2	Multiboard	Bartels Mikrotechnik GmbH
3	1/4-28 Y Connector	Idex
4	Luer-1/4-28 Adaptor	Idex
5	Ferrule	Idex
6	1/4-28-Tubing Adaptor	Idex
7	Inner Tubing	
8	Outer Tubing	Cole-Parmer
9	Connector	microfluidic ChipShop
10	Membrane Pumps	Bartels Mikortechnik GmbH
11	Pump Tubing	Bartels Mikrotechnik GmbH
12	Tank	microfluidic ChipShop
13	Luer Y Connector	Perkin-Elmer
14	Luer-Miniluer-Adaptor	microfluidic ChipShop
15	Plug	microfluidic ChipShop

Supplementary Figure S2. Schematic explosion animation of minimal droplet generator setup. The illustration shows all components necessary for repeating our experiments. The numbers refer to the part list (see *below*). Tubing length is customizable, however, we used lengths of 5 cm of 8) and 3 cm of 7), with enough overlap to ensure no leaking. Not depicted is the collection syringe, which can be any syringe with a Luer lock.

#	Part Description	Supplier	Supplier Name	Supplier Ref # (if applicable)
1	Microfluidic Chip	microfluidic ChipShop	Fluidic 163	10000004
2	Multiboard	Bartels Mikrotechnik GmbH	mp-Multiboard	Part of mp-basic kit
3	1/4-28 Y Connector	Idex	P-512 Y assembly for 1/16" OD tubing, 1/4-28 flat-bottom threads, 0,50 mm thru-hole, 1,7 µl swept volume, PEEK	554-3117
4	Luer-1/4-28 Adaptor	Idex	P-658 adapter, female Luer to female 1/4-28 flat-bottom, 0,05" thru-hole. Red PEEK body	554-1798
5	Ferrule	Idex	Flangeless Ferrule Tefzel™ (ETFE), 1/4-28 Flat-Bottom, for 1/16" OD	P-200
6	1/4-28-Tubing Adaptor	Idex	Flangeless Nut PEEK, Short, 1/4-28 Flat-Bottom, for 1/16" OD	P-235
7	Inner Tubing	Elveflow	Liquid Flows Tygon Tubing 1/16 OD X 0.02" ID	
8	Outer Tubing	Cole-Parmer	Masterflex Transfer Tubing, Platinum-Cured Silicone, 1/32" ID x 3/32" OD; 25 Ft	95802-01
9	Connector	microfluidic ChipShop	Fluidic 331	10000094
10	Membrane Pumps	Bartels Mikrotechnik GmbH	mp6 micropumps	mp-gas+; mp-liq (for aqueous phase content tuning)
11	Pump Tubing	Bartels Mikrotechnik GmbH	mp-t tubing	Supplied with pumps
12	Tank	microfluidic ChipShop	Fluidic 233	10000079
13	Luer T Connector	DirectMed	T Connector with 2 Female Luers + Male Luer Slip	TT-010
14	Luer-Miniluer-Adaptor	microfluidic ChipShop	Fluidic 390	10000063
15	Plug	microfluidic ChipShop	Fluidic 334	10000053

Supplemental Table S1. Parts list of Minimal Droplet Generator Setup. Additionally, a syringe with a Luer tip is needed. Tubings are are interchangeable with other chemically resistant tubing with suitable diameters.

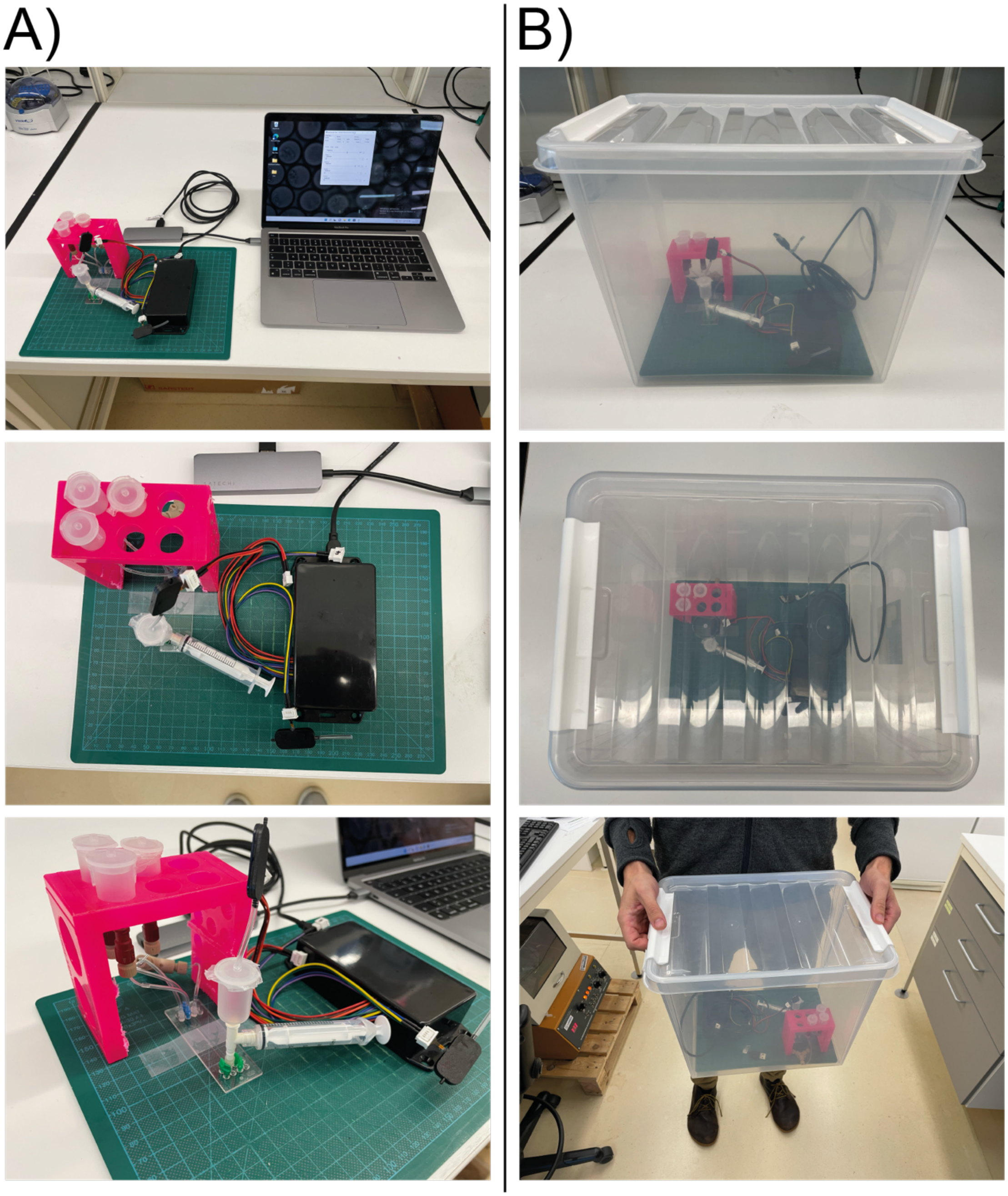

Supplemental Figure S3. The droplet generator has a small footprint and is highly portable. A) shows three images of the setup set up for droplet generation, depicting the microfluidic device, tubing, syringe and reservoirs, the microcontroller and a 13” Macbook Pro (Apple). B) shows three images of the setup (minus the Macbook) in a small carrying box.

Primary Cell Spheroid Production Setup

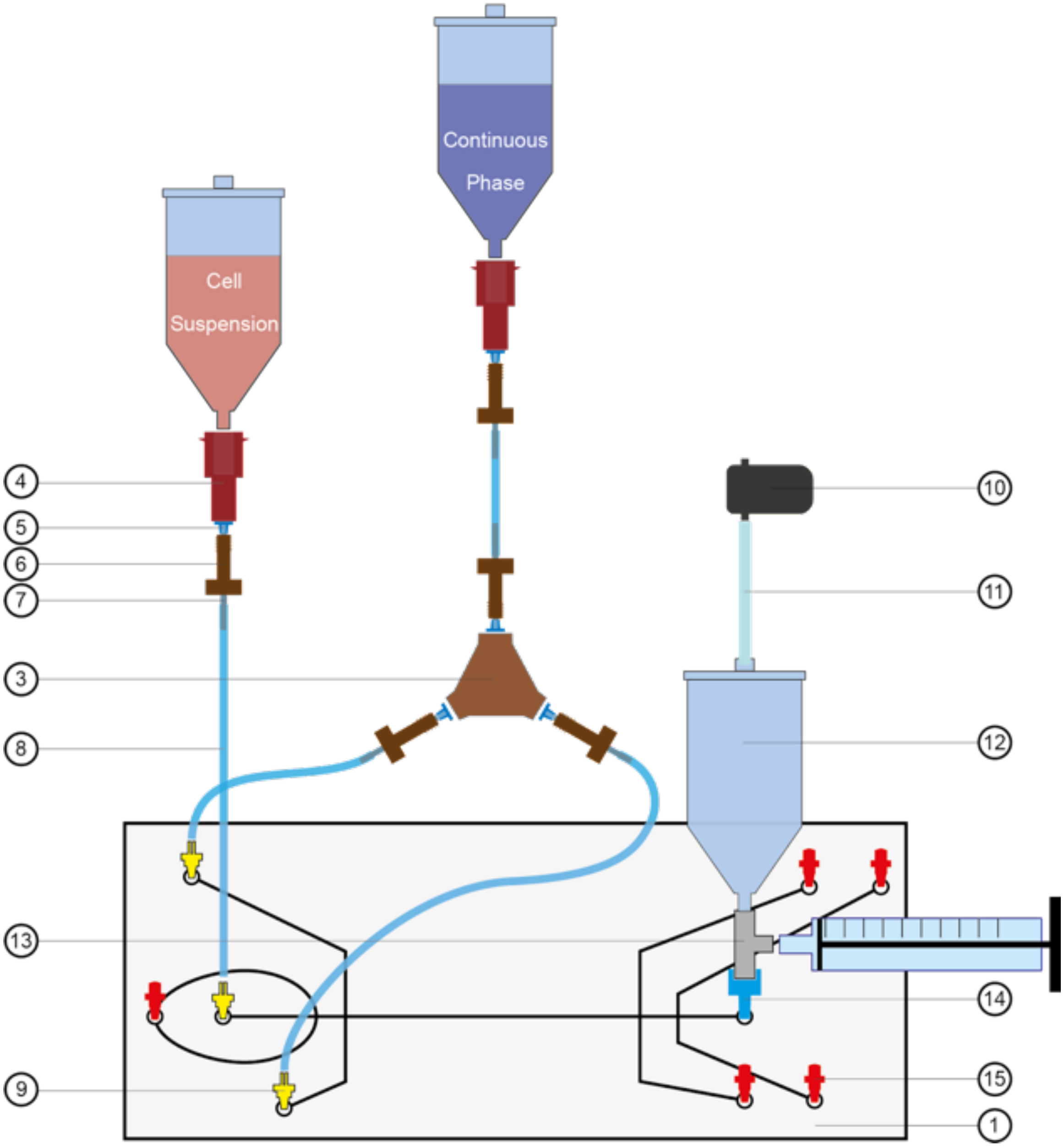

Supplemental Figure S4. Schematic illustration depicting the used configuration for encapsulation of primary hepatocytes. The numbers refer to the parts list above.

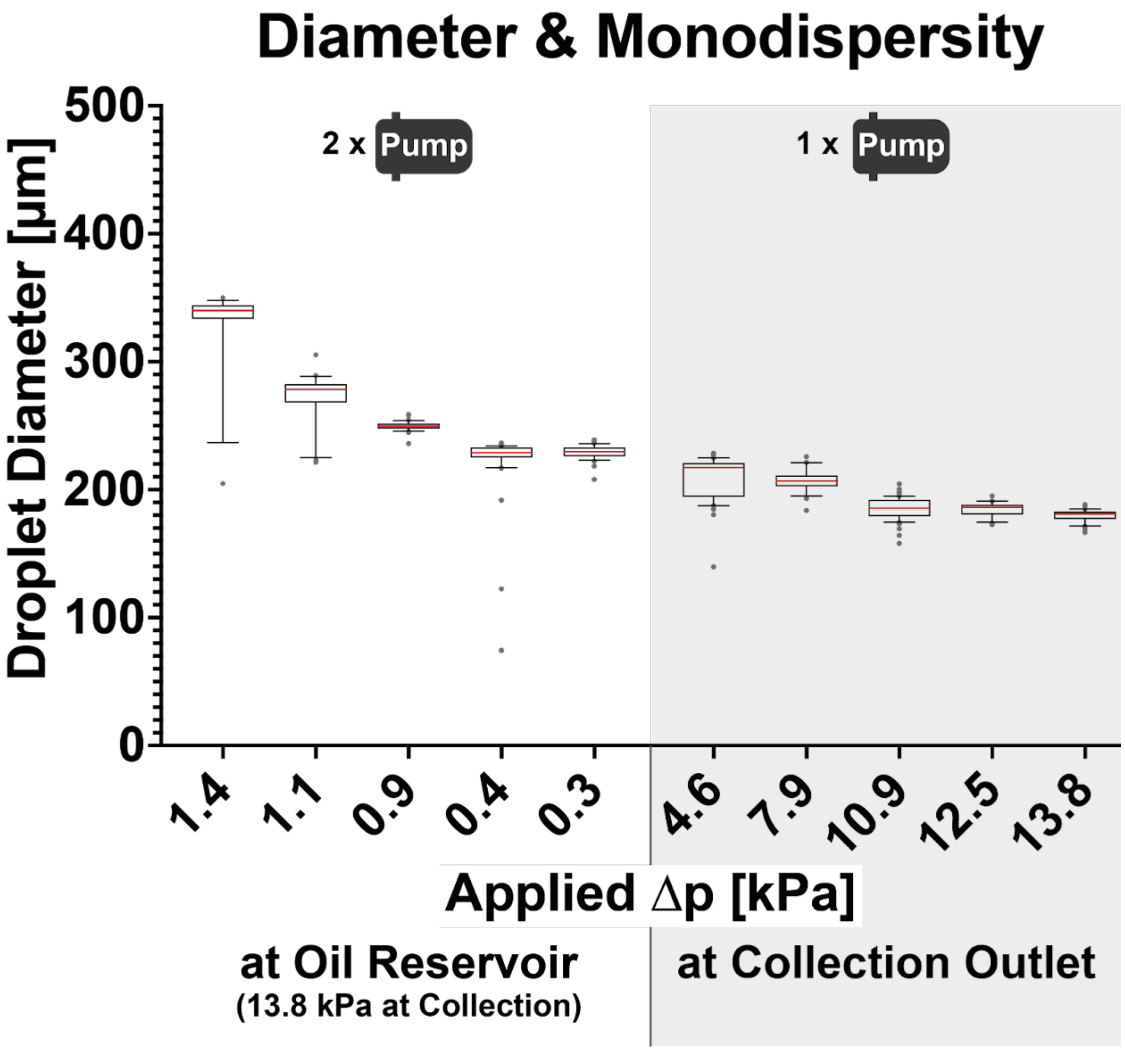

Supplemental Figure S5. Droplet sizes are dependent on applied pressures at the outlets. The measured droplet diameters are monodisperse and decrease with increasing pressure differential in the single pump configuration (gray background). To access larger droplet diameters, a second pump at the oil reservoir might be employed. Red lines signify median, boxes encompass the 25th until the 75th percentile, whiskers indicate 10th until 90th percentile. Dark gray dots show measurements outside of these percentiles.
